# Fall Webworm Host Plant Preferences Generate a Reduced Predation Enemy-Free Space in Its Interaction with Parasitoids

**DOI:** 10.3390/insects16080804

**Published:** 2025-08-04

**Authors:** Lina Pan, Wenfang Gao, Zhiqin Song, Xiaoyu Li, Yipeng Wei, Guangyan Qin, Yiping Hu, Zeyang Sun, Cuiqing Gao, Penghua Bai, Gengping Zhu, Wenjie Wang, Min Li

**Affiliations:** 1 Tianjin Key Laboratory of Conservation and Utilization of Animal Diversity, Tianjin Normal University, Tianjin 300387, China; skypln@tjnu.edu.cn (L.P.); skygwf@tjnu.edu.cn (W.G.); 17658382256@163.com (Z.S.); 17627650324@163.com (X.L.); wzybwyp@126.com (Y.W.); skyqinguangyan@163.com (G.Q.); hypdyouxiang@163.com (Y.H.); skyszy@tjnu.edu.cn (Z.S.); 2 Co-Innovation Center for Sustainable Forestry in Southern China, College of Forestry, Nanjing Forestry University, Long Pan Road, Nanjing 210037, China; 3 Institute of Plant Protection, Tianjin Academy of Agricultural Sciences, Tianjin 300384, China; baipenghua@126.com; 4 Department of Entomology at Washington State University, Pullman, WA 99163, USA; gengping.zhu@wsu.edu (G.Z.); wenjie.wang@wsu.edu (W.W.)

**Keywords:** enemy-free space, tritrophic interactions, parasitoid, *Hyphantria cunea*, *Chouioia cunea*, GC-EAD, tridecane

## Abstract

Plants and insects are developing strategies to avoid each other’s defense systems. Host plants may release volatile compounds to attract the natural enemies of herbivores; insect pests may also select host plants that are deterrent to natural enemies to avoid such predation. Here we investigated whether the host plant preference of *Hyphantria cunea* correlates with the attractiveness of these plants to *Chouioia cunea*, a parasitoid wasp that serves as the primary natural enemy of *H. cunea*. We found *Morus alba* was the preferred host plant for female *H. cunea*. Although *M. alba* provided suboptimal nutritional value for *H. cunea* growth and development compared to other plants, it attracted fewer *C. cunea* relative to alternative host plants. Gas chromatography–mass spectrometry (GC–MS) coupled with gas chromatography–electroantennographic detection (GC-EAD) analysis identified six distinct compounds among the herbivore-induced plant volatiles (HIPVs) produced following *H. cunea* feeding. Notably, *M. alba* was the sole plant species that did not emit tridecane. These results suggest that *H. cunea* utilizes *M. alba* as a reduced predation enemy-free space, thereby minimizing parasitization by *C. cunea*. Our research emphasizes the importance of considering adaptive responses of herbivores within the context of multi-trophic relationships, rather than solely focusing on optimizing herbivore growth on the most nutritionally suitable plant host.

## Simple Summary

The tri-trophic interactions among the fall webworm (*Hyphantria cunea*), its host plants, and its parasitoid wasp (*Chouioia cunea*) were investigated, with particular focus on the correlation between *H. cunea*’s host plant preference and the attractiveness of these plants to *C. cunea*. It was found that *Morus alba*, despite providing suboptimal nutritional quality for *H. cunea* larvae, became the most preferred host plant due to its significantly reduced attraction to *C. cunea* compared to alternative host species. Through volatile analysis, *M. alba* was identified to lack tridecane emission, a key semiochemical that attracts *C. cunea*. These results demonstrate adaptive host selection behavior in *H. cunea* that minimizes parasitism risk, providing novel insights for refining biological control strategies against this invasive species.

## 1. Introduction

Plants and insects have coexisted for over 350 million years, during which both have developed strategies to avoid each other’s defense systems. For instance, host plants may release volatile compounds that attract the natural enemies of herbivores by offering sustenance and shelter to these natural enemies, and insect pests may also select host plants that are deterrent to natural enemies to avoid such predation (i.e., enemy-free space). There are numerous physical, physiological, and behavioral mechanisms that can support ‘enemy-free space’ [1,2,3]. For instance, gall wasps are defended against natural enemies by rapidly changing gall morphology [4,5,6]. Herbivores may also develop behavioral preferences to consume or oviposit on host plants that are nutritionally sub-optimal but are less frequented by their natural enemies. Consequently, herbivores preferring these less optimal plant species may experience enhanced survival compared to more nutritious hosts on which the herbivore is more susceptible to parasitization [2,7,8,9,10].

Research suggests that the influence of host plants on the risk of attack by parasitoids is an important selective force in the evolution of herbivore diet breadth [11]. For example, larvae of the generalist arctiid moth *Platyprepia virginalis* experienced 83% parasitism by tachinid flies on hemlock (*Conium maculatum*), whereas <50% of the caterpillars collected from lupin (*Lupinus albus*) were parasitized [12]. Another example of a generalist herbivore feeding on suboptimal plants to reduce parasitization is the arctiid *Grammia geneura*: its larvae tend to feed on plant species that, although nutritionally suboptimal, may offer chemical compounds that enhance their survival following parasitization [4]. All these examples suggest that host plant preferences are governed not only by nutritional quality but also by the third trophic level, natural enemies [2,3,13,14].

The fall webworm *Hyphantria cunea* (Drury 1773) (Lepidoptera: Erebidae) is a notorious invasive species. In China, since its introduction in 1979, the moth has spread into numerous provinces and cities, including Liaoning, Shandong, Anhui, Shanxi, Hebei, Henan, Shanghai, Tianjin, and Beijing, and continues to expand its range [15,16]. The larvae could feed on >600 host plant species, with about 100 found in China [17]. The parasitoid wasp *Chouioia cunea* Yang 1989 (Hymenoptera: Eulophidae) [18] is an endoparasitic chalcid wasp indigenous to China that parasitizes *H. cunea*. A single *H. cunea* pupa may yield between 145 and 365 adult wasps, with a remarkably high percentage (98–99%) of emerging wasps being female [19]. In China, *C. cunea* is widely used in *H. cunea* biological control programs [20,21,22,23].

Although *H. cunea* can subsist on hundreds of plant species, the larvae exhibit preferences for certain host plants [24,25,26]. It remains unclear whether the nutritionally optimal plants for *H. cunea* correspond to its most preferred hosts; it is also not clear whether the preferred host plants are most attractive to both *H. cunea* and its parasitoid *C. cunea*. Infochemicals provide important cues for insects to locate and recognize their hosts and avoid enemies [27,28,29]. Some work has focused on plant–herbivorous insect interactions, such as the role of stored plant volatiles on pests [30]; for example, the phenolic acids in the leaves of hazel (*Corylus* spp.) have an allelopathic effect on *Myzocallis coryli* [31], and granary weevils are attracted or repelled by different concentrations of cereal volatiles [32].

Other researchers have shown that chemicals from *H. cunea* pupae or its by-products (e.g., frass) could induce the arrestment of *C. cunea* females [33], and the response of parasitoid *Holepyris sylvanidis* to host-associated volatiles can also improve parasitization success [34]. However, these works focused on the paired interactions between *H. cunea* and its parasitoids but rarely analyzed the tritropic interactions among host plants, insects, and natural enemies from the perspective of infochemical communication. In this study, the adaptive responses of herbivores were considered in the context of multi-trophic relationships, and the chemical ecology and behavior of both the herbivore and the parasitoid were investigated. Our research could contribute to our understanding of *H. cunea* invasion spreading, and results provided here could also improve biological control efficiency by promoting *C. cunea* parasitization.

## 2. Materials and Methods

### 2.1. Insects Rearing

*C. cunea* parasitoid wasps were obtained from the Natural Enemy Breeding Center of Luohe Central South Forestry (Henan, China) in 2012. *Antheraea pernyi* (Lepidoptera: Saturniidae) was the substitute host of *C. cunea*, obtained from the Benxi Tussah Breeding Base (Liaoning, China). The *H. cunea* eggs were obtained from the Chinese Academy of Forestry in 2020, where they were reared on an artificial diet after hatching. The hatched larvae were then separately reared till pupation on the leaves of mulberry (*Morus alba* L.), peach (*Amygdalus persica* L. var. persica f. duplex Rehd), empress tree (*Paulownia tomentosa*), tree of heaven (*Ailanthus altissima* (Mill.) Swingle), weeping willow (*Salix babylonica*), Korean aspen (*Populus davidiana*), Chinese ash (*Fraxinus chinensis*), and Old World sycamore (*Platanus orientalis* Linn.). The leaves of these eight plant species were collected on the day of the experiment at the Tianjin Normal University campus.

### 2.2. Larvae Performance on Host Plants

To test larvae performances on different host plants, three sets of 100 hatched larvae were fed with eight plant species’ leaves separately. As the body length and weight of *H. cunea* varied very little before the fourth instar, only the data after the 4th instar were collected. When the larvae reached the 4th instar (from the 12th day after hatching), 10 larvae were randomly selected from each cage every 2 days, and their body length and body weight were measured. The pupation rate was calculated after all the larvae pupated. The cages were kept in an incubator at 25 °C and 70% relative humidity (RH) with a 14:10 light: dark cycle.

### 2.3. Volatiles Collection

Before collecting volatiles, the leaves were immersed in water for two hours to remove biological contaminants and pesticides, after which they were allowed to air-dry naturally. Two groups were considered for dynamic volatile collections: the first group remained unexposed to herbivores prior to headspace collection; and the second was exposed to 5th instar larvae that had been deprived of food for 12 h. Following 6 h of leaf consumption by the larvae, the insects and their frass were removed, and headspace collection commenced.

For collecting volatiles for chromatography analysis, twenty grams of leaves were placed in each specialized 1 L glass conical flask. Purified room air, filtered through activated charcoal, was continuously passed through the system at a flow rate of 600 mL-min^−1^. Volatile compounds were trapped using glass tubes containing 100 mg of Porapak™ Q (Waters Corporation, Shanghai, China), retained between silanized glass wool plugs, with an effluent flow rate of 500 mL-min^−1^. The consistent positive pressure within the chamber ensured that no ambient air infiltrated the chamber. Volatile compounds were collected in the Porapak Q traps for 6 h and subsequently eluted with 1 mL of freshly distilled hexane. Through nitrogen evaporation, the sample was concentrated to 100 µL and preserved at −20 °C for subsequent chromatographic analysis.

### 2.4. Gas Chromatography and GC-MS

Volatiles were separated and quantified by Agilent GC 7890A, equipped with a DB-5MS column (30 m × 250 μm × 0.25 μm, Agilent Technologies, Santa Clara, CA, USA), with splitless injection (250 °C). The oven temperature was maintained at 50 °C for 1 min, then programmed at 4 °C min^−1^ to 150 °C and subsequently held at this temperature for 1 min, and then programmed at 10 °C min^−1^ to 230 °C, followed by a 10 min hold. The carrier gas was ultra-high purity helium (1.2 mL-min^−1^).

The electrophysiologically active gas chromatography peaks were analyzed using an Agilent 7200 Q-TOF mass spectrometer (EI, 70 eV, source temperature 250 °C, *m*/*z* 50–650). Tentative identification of the *C. cunea* EAD-active compounds was based on (1) Kovats indices, (2) electron ionization mass spectra, and (3) comparison with authentic chemicals.

### 2.5. Gas Chromatography-Electroantennography

The electrophysiological preparation was initiated by excising a single antenna from a 1-day-old mated *C. cunea* female using microsurgical scissors under a magnifying lens. A glass capillary (0.5 mm inner diameter) filled with 0.1 M KCl solution was used as an electrode. The reference electrode was attached to the base of the isolated antenna, and the recording electrode was connected to the cut tip of the antenna. Chlorinated silver–silver chloride junctions were used to ensure electrical contact between the electrodes and input of the preamplifier.

The analog signal captured by a probe (INR-II; Syntech, Kirchzarten, Germany) was processed using a data acquisition controller (IDAC-232; Syntech) and was analyzed subsequently using customized software (GC-EAD 1.2.5, Syntech). For GC-EAD coupling, the GC column effluent was simultaneously split to the antennal preparation and flame ionization detector, FID. The collected volatiles were separated by GC (6820, Agilent Technologies, Stockport, UK) using a 30 m × 0.32 mm × 0.25 μm ID HP-5MS column (Agilent). Carrier gas flow (helium) and temperature programming followed the previously described GC-MS method. Both EAD amplifier output and the FID were monitored simultaneously and analyzed using Syntech software. A peak was deemed electrophysiologically active if it elicited responses on three or more independent antennal replicates.

### 2.6. Chemicals

Synthetic standards necessary for the confirmation of identity and behavioral bioassays were obtained from J&K Chemical Co. (Beijing, China) (nonadecane 99.5%, CAS 629-92-5; hexadecane 99%, CAS 544-76-3) and Aladdin Co. (Shanghai, China) (undecane ≥ 99%, CAS 1120-21-4; heneicosane > 99%, CAS 629-94-7; tridecane 99%, CAS 629-50-5; tetradecane 99%, CAS 629-59-4).

### 2.7. Behavioral Assays

#### 2.7.1. Responses of Mated Female *H. cunea* to Different Plants

Due to the potential impact of plant leaf consumption on *H. cunea* behavior, *H. cunea* specimens were provided with an artificial diet. Individual leaves (not previously infested by *H. cunea*) of approximately equivalent mass (5 g) from eight plant species were cleansed with water and allowed to air-dry naturally. Two leaves were affixed to each side of a cage (30 × 30 × 30 cm) using adhesive tape. The experiments were performed in a darkened room illuminated by red light. Fifteen mated female moths were placed in three Petri dishes, which were positioned in the center of the cage. The Petri dishes were left uncovered, and moth behavior was observed for a duration of 30 min. For each of the eight replications, fresh plant leaves and new *H. cunea* females were utilized.

#### 2.7.2. Responses of 1-Day-Old Mated *C. cunea* Females to Various Odor Sources

Both Y-tube (binary choice) and four-arm olfactometers were employed to assess the behavioral preferences of 1-day-old mated *C. cunea* females to various chemical stimuli. The Y-tube olfactometer consisted of a common glass tube (11.5 cm long × 2.2 cm diameter) and two lateral glass arms (7.5 cm long × 2.2 cm diameter). A purified air stream (200 mL-min^−1^) was generated by an air pump through activated charcoal and doubly distilled deionized water. The experiment was conducted in a dark room. A lamp (25 W, 250 lx) was positioned 55 cm above the olfactometer for illumination.

The four-arm olfactometer (QT-WII01, Channel Technology Co., Beijing, China) was constructed with an acrylic chamber (10 × 10 cm) partitioned into four zones. The top of the chamber was covered with a glass plate to prevent insect escape. The experiment was conducted in a dark room. A light bulb was positioned on top of the arena, such that the amount of illumination (∼250 lx) was consistent for all arms.

The experiments involved the following comparisons, all examining the attraction of *C. cunea* (i) to different plant leaves before and after they were consumed by *H. cunea* larvae; (ii) to the eight plant leaves after they were consumed by *H. cunea* larvae; (iii) to single compounds at different doses versus hexane (carrier solvent); and (iv) to different synthetic blends (see detailed methods below).

(i) In total, 5 g of uncontaminated plant leaves were positioned in one arm of the Y-tube olfactometer, while 5 g of leaves from the same plant, consumed by *H. cunea*, were placed in the other arm. The leaves were separated using 70 mesh absorbent cotton gauze to prevent physical contact. Forty mated female *C. cunea* (1-day-old) were released at the base of the common arm of the Y-tube and monitored for a maximum of 5 min. Females failing to exhibit orientation behavior within 5 min were excluded from the analysis. Females that traversed 1 cm beyond the Y-junction and remained there for at least 10 s were recorded to have made a selection. Following each assay, the tube was cleansed with soap, water, hexane, and air dried, and its orientation was reversed. Five independent biological tests were performed for each odor source. Each parasitoid was utilized only once. All bioassays were conducted at 25 °C and 60% relative humidity (RH). The female response percentage was calculated as follows: Rs = 100% × (parasitoids that selected the treatment arm)/(parasitoids that selected the treatment arm + parasitoids that selected the control arm).
(ii) The responses of *C. cunea* to the eight different plant leaves after they were consumed by *H. cunea* were investigated using a four-arm olfactometer. The experiment was conducted as follows: group 1: the attraction of four plants to the wasps was compared; group 2: attraction to the other four plants was compared; group 3: the two most attractive plants in group 1 were compared with the two most attractive plants in group 2; and group 4: the two least attractive plants in group 1 were compared with group 2 again. Leaves (5 g) consumed by *H. cunea* were placed inside the chambers of four randomly selected arms of the olfactometer. Forty 1-day-old mated female *C. cunea* were introduced in the center of the arena, and their selection of the four arms was recorded. A selection was defined as entering one of the four arms and remaining there for at least 10 s. The odor sources in the olfactometer were replaced, and their positions were altered at every assay. Additionally, the entire arena and odor chambers were cleansed with soapy water followed by hexane and then air-dried. Each parasitoid was utilized only once. Nine independent biological tests were conducted, and the bioassays were performed at 25 °C and 60% RH.
(iii) For chemical standard testing, test compounds at various concentrations (10, 100, 1000, and 10,000 ng/μL, 10 μL) were applied to filter papers. After a 20 s period to allow for solvent evaporation, the filter papers were positioned in one arm of the Y-tube olfactometer. A corresponding filter paper with 10 μL of hexane was placed in the opposite arm (solvent control). The bioassay procedures with *C. cunea* were conducted as previously described.
(iv) The responses of *C. cunea* to 10 μL of synthetic blends of EAD-active compounds were examined using a four-arm olfactometer. Filter papers containing synthetic blends of EAD-active compounds (10 ng/uL for each component), synthetic blends (excluding tridecane), and hexane were positioned within the chambers of three randomly selected arms of the olfactometer. The fourth arm received 10 μL hexane and functioned as a solvent control. The bioassay procedures with *C. cunea* were conducted as previously described.

#### 2.7.3. Responses of Female Adult *H. cunea* to Different Compounds

The olfactory responses of mated *H. cunea* females to various compounds were examined utilizing a Y-tube olfactometer. In group 1, a filter paper containing 10 μL tridecane (10 ng/μL) was positioned in one arm of the Y-tube olfactometer, while hexane (solvent control) was placed in the other arm. For group 2, a filter paper with a synthetic blend of EAD-active compounds excluding tridecane was situated in one arm of the Y-tube olfactometer, and 10 μL of the identical synthetic blend including tridecane (10 ng/μL for each component) was positioned in the other arm. The bioassay procedures for *C. cunea* were conducted in accordance with the aforementioned protocol.

### 2.8. Statistical Analysis

One-way analysis of variance followed by a Tukey post hoc multiple comparison test was used to compare (i) the performance of *H. cunea* larvae (body length, body weight, and pupation rates) fed on different plant species leaves (Section 2.2); (ii) the preferences of mated female *H. cunea* to different host plants (Section 2.7.1); (iii) the preferences of *C. cunea* to different plant leaves that had been fed upon by *H. cunea* in a four-arm olfactometer (Section 2.7.2, ii); and (iv) the responses of *C. cunea* to different synthetic blends of EAD-active compounds in a four-arm olfactometer (Section 2.7.2, iv). All behavioral responses of female *C. cunea* in the Y-tube olfactometer were analyzed using the Wilcoxon rank sum test, including (i) the responses of *C. cunea* to the leaves of eight plant species before and after *H. cunea* feeding (Section 2.7.2, i); (ii) the responses of *C. cunea* females to different concentrations of tridecane (Section 2.7.2, iii); and (iii) the responses of *H. cunea* to hexane control and tridecane (Section 2.7.3). All analyses were performed in R 4.3.0 (www.r-project.org).

## 3. Results

### 3.1. Growth of H. cunea Fed Different Plant Leaves

**Figure 1:**
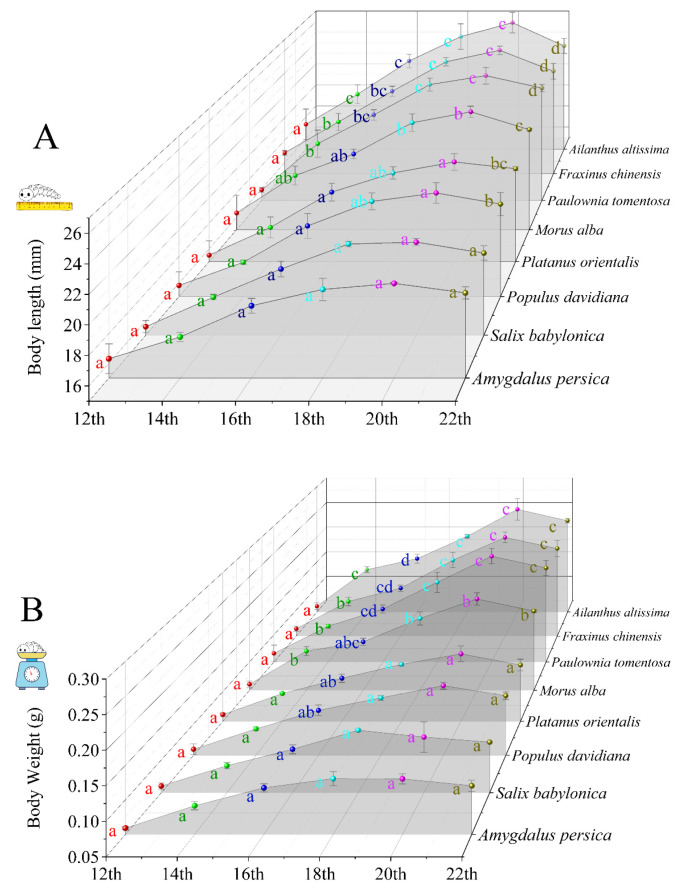
The body length (**A**) and body weight (**B**) of *H. cunea* larvae fed on the leaves of eight plant species. The results are expressed as the mean ± standard deviation. Data within each date (with the same color) that do not share common letters are significantly different as determined by a one-way ANOVA followed by Tukey’s HSD test (*p* < 0.05).

**Figure 2:**
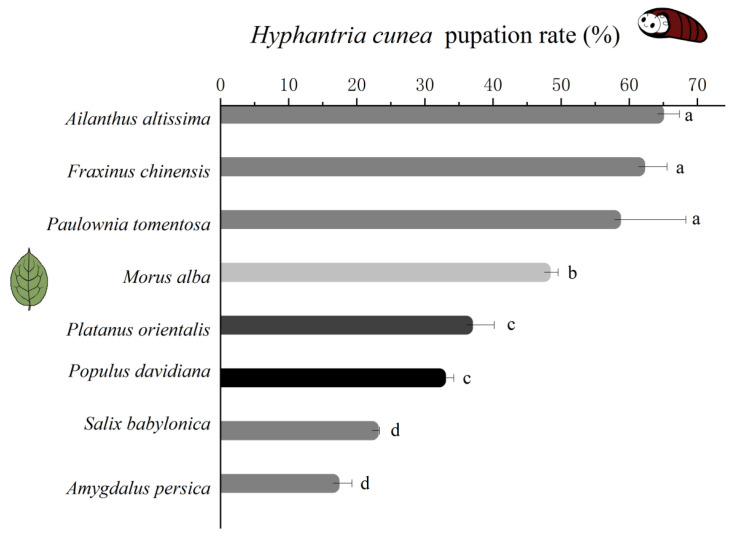
The pupation rate of *H. cunea* larvae fed on the leaves of eight plant species. The results are expressed as the mean ± standard deviation. The different letters are significantly different as determined by a one-way ANOVA followed by Tukey’s HSD test (*p* < 0.05).

Initially, no significant differences were observed in the length and weight of larvae feeding on the leaves of different plants (*p* > 0.05, 12th day after hatching). Subsequently, *H. cunea* specimens that consumed the leaves of *A. altissima*, *F. chinensis*, and *P. tomentosa* exhibited greater length and weight compared to those feeding on *M. alba*, *P. davidiana*, *P. orientalis*, *A. persica*, and *S. babylonica* (*p* < 0.05) (Figure 1). For example, on the 18th day after hatching, *H. cunea* larvae feeding on *A. altissima* exhibited optimal growth performance, with a mean body length of 25.47 ± 1.19 mm and body weight of 0.20 ± 0.00 g. Those consuming M. alba leaves measured 23.77 ± 0.72 mm in body length and 0.18 ± 0.01 g in weight. Larvae reared on *A. persica* showed the smallest dimensions, with a mean body length of 20.97 ± 0.76 mm and weight of 0.13 ± 0.01 g. Pupation rate demonstrated a similar trend, with significantly higher pupation rates observed in *H. cunea* specimens that consumed the leaves of *A. altissima*, *F. chinensis*, and *P. tomentosa* compared to those that fed on other plant species (*p* < 0.05) (Figure 2). These findings suggest that *A. altissima*, *F. chinensis*, and *P. tomentosa* provided superior nutritional content for the growth and development of *H. cunea* compared to *M. alba* and the other plant species examined.

### 3.2. H. cunea Preference of Eight Plant Species

**Figure 3:**
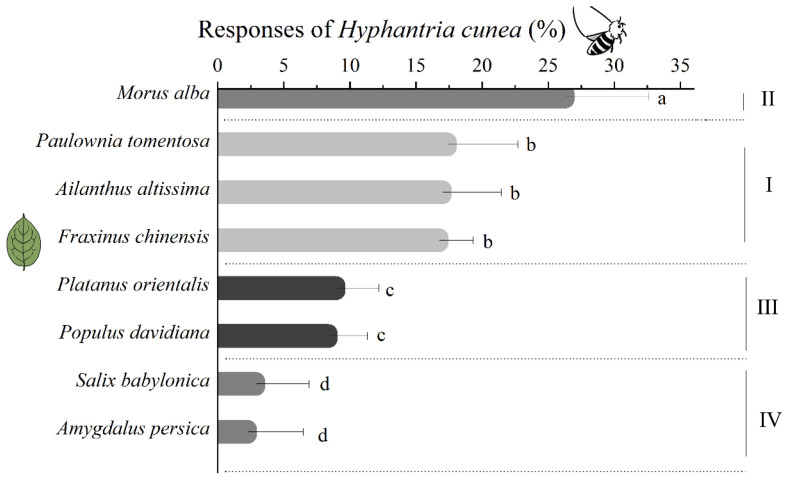
Mated female *H. cunea* selection on eight host plants. The results are expressed as mean ± standard deviation. The different letters are significantly different as determined by a one-way ANOVA followed by Tukey’s HSD test (*p* < 0.05). The capital Roman numerals on the right indicate the degree of growth of *H. cunea* on different plants, which is based on the results in Figure 1 and Figure 2.

Mated female *H. cunea* selected plants inconsistent with larval growth parameters (Figure 1 and Figure 2). The preference hierarchy was as follows: 1, *M. alba*; 2, *P. tomentosa*, *A. altissima*, and *F. chinensis*; 3, *P. orientalis* and *P. davidiana*; and 4, *S. babylonica* and *A. persica* (*p* < 0.05) (Figure 3). The preferred host plant of female *H. cunea* (*M. alba*) is not optimal for larval growth and development.

### 3.3. Responses of C. cunea to Leaves Before and After Feeding by H. cunea

**Figure 4:**
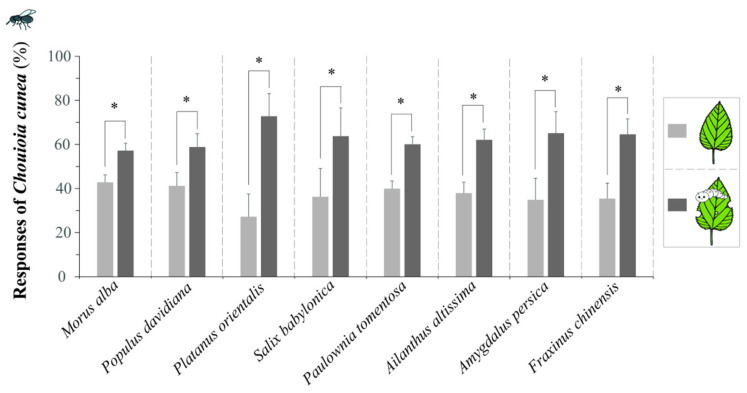
The responses of *C. cunea* to eight plant species leaves before and after *H. cunea* feeding on the leaves in a 2-choice Y-tube olfactometer. Light gray bars represent the responses of *C. cunea* to intact (unfed) plant leaves, whereas the black bars represent the responses to leaves after feeding by *H. cunea*. The results are expressed as the mean ± standard deviation. * indicates a significant difference compared with the respective control (intact) group as analyzed using the Wilcoxon rank sum test by R (*p* < 0.05).

The leaves of all eight plant species exhibited significantly higher attractiveness to *C. cunea* after *H. cunea* feeding compared to before feeding (*F. chinensis*, *p* = 0.009; *P. orientalis*, *p* = 0.008; *A. altissima*, *p* = 0.009; *P. davidiana*, *p* = 0.009; *M. alba*, *p* = 0.009; *P. tomentosa*, *p* = 0.009; *A. persica*, *p* = 0.009; *S. babylonica*, *p* = 0.009) (Figure 4).

### 3.4. Comparison of Responses of C. cunea to Different Plant Leaves Fed upon by H. cunea

**Figure 5:**
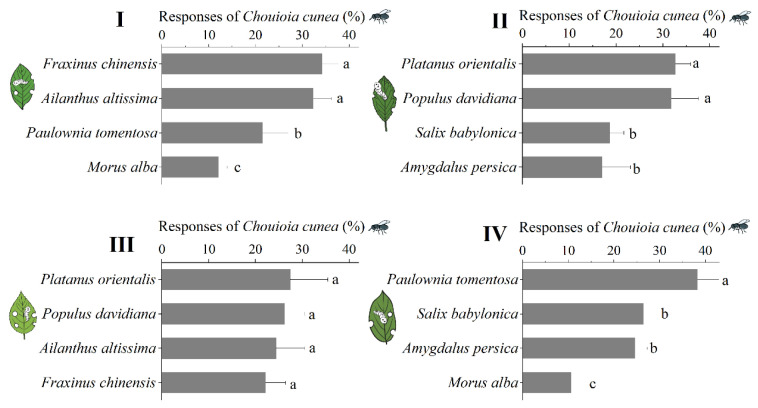
The responses of *C. cunea* to leaves of different plant species that had been fed upon by *H. cunea*. Group **I**: response to 4 plants; group **II**: responses to the other 4 plants; group **III**: responses to the most attractive 2 plants from groups 1 and 2; group **IV**: responses to the least attractive 2 plants from groups 1 and 2. Forty mated female *C. cunea* (1 day old) were released each time. Each parasitoid was used only once. Nine independent biological tests were run. The results are expressed as the mean ± standard deviation. Plants stripes within a group that do not share common letters are significantly different as determined by a one-way ANOVA followed by Tukey’s HSD test (*p* < 0.05).

The four-arm olfactometer was utilized to assess *C. cunea* preferences for different plant leaves that had been subjected to *H. cunea* herbivory. Four experimental groups were established to compare the attraction of *C. cunea* to various plant species (Figure 5):

Group I: *F. chinensis* (34.21%) and *A. altissima* (32.29%) elicited greater attraction of *C. cunea* compared to *P. tomentosa* (21.45%) and *M. alba* (12.05%).

Group II: *P. orientalis* (32.67%) and *P. davidiana* (31.75%) demonstrated higher attraction of *C. cunea* in comparison to *A. persica* (16.99%) and *S. babylonica* (18.60%).

Group III: The four most attractive plants in groups I and II were subjected to comparative analysis. No statistically significant differences were observed in the attractiveness of *F. chinensis*, *A. altissima*, *P. orientalis*, and *P. davidiana* (*p* = 0.395).

Group IV: The four least attractive plants in groups I and II were compared. P. tomentosa (38.26%) exhibited significantly higher attraction of *C. cunea* compared to *A. persica* (24.63%) and *S. babylonica* (26.46%), while *M. alba* attracted the fewest *C. cunea* (10.65%).

Consequently, the attractiveness to *C. cunea* of host plants subjected to *H. cunea* herbivory can be categorized into four levels. *F. chinensis*, *A. altissima*, *P. orientalis*, and *P. davidiana* exhibited the highest attractiveness to *C. cunea. P. tomentosa*, *A. persica*, and *S. babylonica* demonstrated significantly lower attractiveness compared to the aforementioned five plant species. *M. alba*, which is the most preferred host of *H. cunea*, exhibited the least attractiveness to *C. cunea*.

### 3.5. Chemical Analysis

The leaf volatiles of eight different plant species before and after *H. cunea* herbivory were analyzed using GC-EAD and GC-MS techniques. Six compounds (undecane, tridecane, tetradecane, hexadecane, nonadecane, and heneicosane) present in the plant leaf volatiles elicited EAD responses in the antennae of *C. cunea* females. Among the eight plants examined, tridecane was not detected solely in *M. alba* (Table 1).

### 3.6. Olfactometer Bioassays

#### 3.6.1. Tridecane vs. Hexane

**Figure 6:**
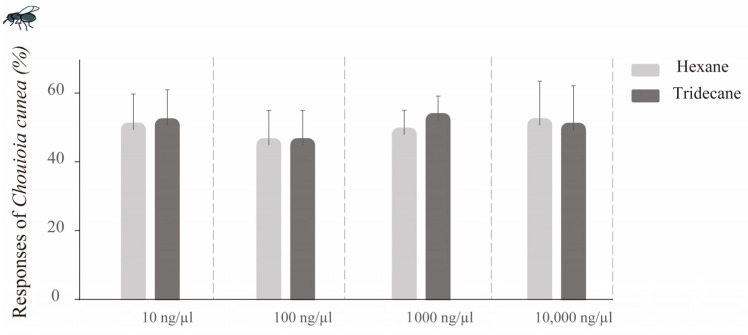
The responses of *C. cunea* females to various doses of tridecane vs. hexane control in 2-choice Y-tube olfactometer. The results are expressed as the mean ± standard deviation. Compared with hexane, none of the concentrations of tridecane elicited responses from *C. cunea*.

As tridecane, a HPIV in seven of the eight host plants, was not detected solely in *M. alba* leaves, the effect of tridecane on the wasp was evaluated utilizing a two-choice Y-tube olfactometer. In comparison with hexane, none of the concentrations of tridecane elicited significant attraction or repellency in the wasp (10 ng/µL: *p* = 0.288; 100 ng/µL: *p* = 0.307; 1000 ng/µL: *p* = 0.353; 10,000 ng/µL: *p* = 0.964) (Figure 6).

#### 3.6.2. Responses of *C. cunea* to Synthetic Blends of EAD-Active Compounds

**Figure 7:**
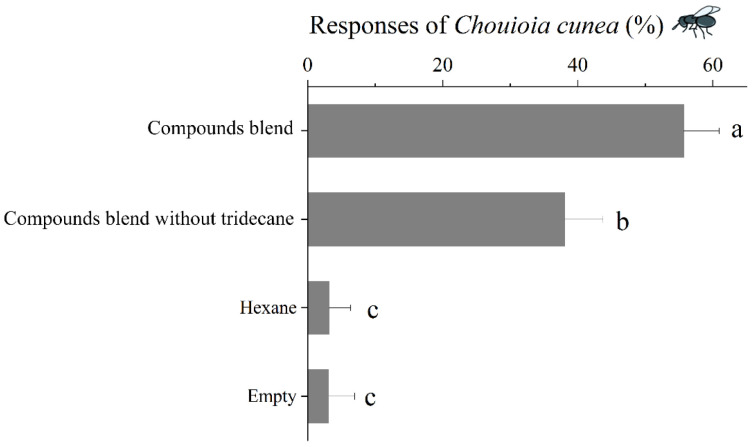
The responses of *C. cunea* to different synthetic blends of EAD-active compounds in four-arm olfactometer. The results are expressed as the mean ± standard deviation. Means with different letters are significantly different as determined by a one-way ANOVA followed by Tukey’s HSD test (*p* < 0.05).

Employing a four-arm olfactometer, the blank arm and the hexane control attracted minimal wasps, whereas the synthetic blend of hydrocarbons demonstrated high attractiveness. However, the removal of tridecane from the blend resulted in a significant reduction in attraction to the blend (*p* < 0.05) (Figure 7).

#### 3.6.3. Responses of *H. cunea* Females to Different Compounds

**Figure 8:**
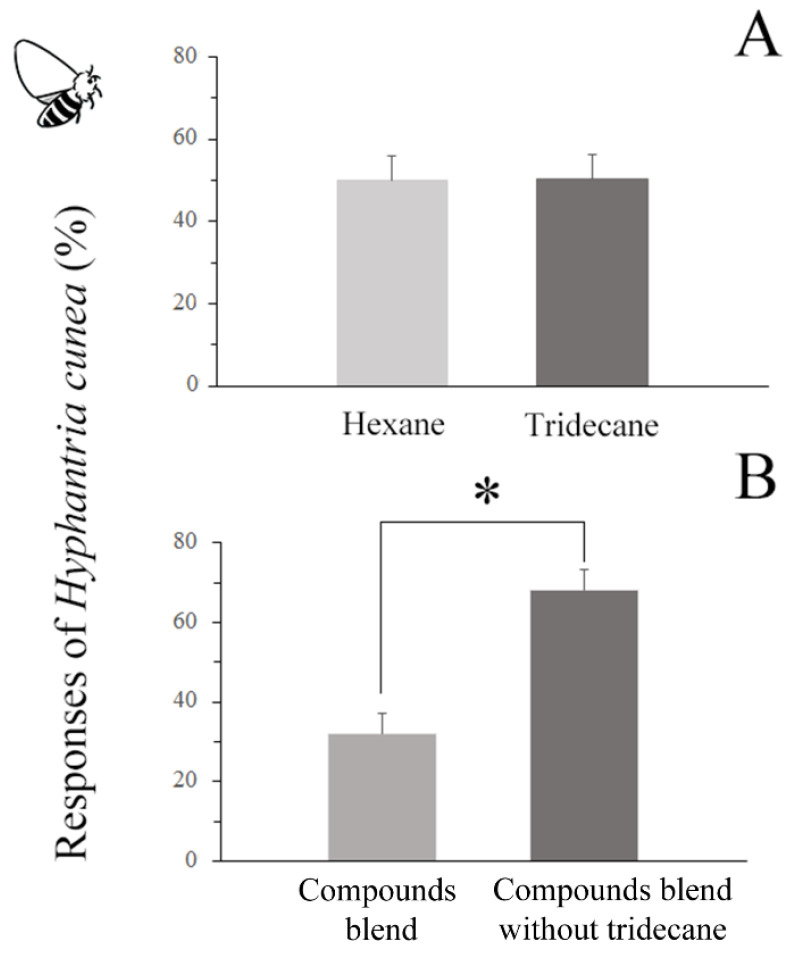
(**A**) Responses of *H. cunea* to hexane control and tridecane in 2-choice Y-tube olfactometer. (**B**) Responses of *H. cunea* to synthetic blends with and without tridecane in 2-choice Y-tube olfactometer. The results are expressed as the mean ± standard deviation. * indicates a significant difference compared with the respective control group as analyzed using the Wilcoxon rank sum test by R (*p* < 0.05).

Similarly to *C. cunea* females, *H. cunea* females exhibited no significant preference for tridecane over the hexane control (*p* = 0.965) (Figure 8A). However, while *C. cunea* females demonstrated a preference for the hydrocarbon blend with tridecane, *H. cunea* females significantly preferred the synthetic blend without tridecane (*p* < 0.0001) (Figure 8B).

## 4. Discussion

Based on the ‘enemy-free space’ hypothesis, insects may exhibit a preference for host plants that are nutritionally suboptimal for growth and development but are less frequented by their natural enemies, thereby reducing exposure to predators and parasitoids. In this study, *H. cunea* larvae were reared on leaves of eight host plant species, including *M. alba*, *A. persica*, *P. tomentosa*, *A. altissima*, *S. babylonica*, *P. davidiana*, *F. chinensis*, and *P. orientalis.* Previous findings indicated that *M. alba*, *P. davidiana*, *P. tomentosa*, *A. altissima*, *F. chinensis*, and *P. orientalis* were the preferred host plants of *H. cunea* [35], whereas *S. babylonica* and *A. persica* were less preferred host plants [36].

Our study results demonstrated that *H. cunea* larvae achieved greater body length, body mass, and pupation rate when feeding on the leaves of *A. altissima*, *F. chinensis*, and *P. tomentosa* compared to the leaves of *M. alba*, followed by *P. davidiana*, *P. orientalis*, *A. persica*, and *S. babylonica*. Thus, our assays support the hypothesis that *A. altissima*, *F. chinensis*, and *P. tomentosa* provided superior nutrition for the growth and development of *H. cunea* compared to *M. alba* and the other plant species.

In contrast to their growth performance on the various plant species, *H. cunea* females exhibited the highest attraction to *M. alba*, followed by *P. tomentosa*, *A. altissima*, *F. chinensis*, *P. orientalis*, *P. davidiana*, *S. babylonica*, and *A. persica*. Yu (2016) [37] observed that *H. cunea* oviposited more eggs on *M. alba* than on 11 other host plant species, which is consistent with our preference results.

The preferred host plant of female *H. cunea* (*M. alba*) is not its optimal nutritional host for growth and development. Conversely, the optimal nutritional hosts (*A. altissima*, *F. chinensis*, and *P. Tomentosa*) are not the most preferred host plants. A plausible explanation for this discrepancy between behavioral preference and physiological performance is the influence of the third trophic level—natural enemies that exert selection pressure on behavioral preferences.

Herbivore-induced plant volatiles (HIPVs) play crucial roles in tri-trophic interactions [3,38]. Therefore, we initially investigated the HIPVs released by plants in response to *H. cunea* feeding that attract the parasitoid *C. cunea*. *C. cunea* exhibited significantly higher attraction to all eight plant species after feeding by *H. cunea* compared to before feeding. Thus, it is evident that herbivory by *H. cunea* induces the plants to release HIPVs that subsequently attract the natural enemy *C. cunea*.

Subsequently, we compared the attraction capabilities of *C. cunea* to various HIPVs. *C. cunea* exhibited the highest attraction to *H. cunea*-fed *F. chinensis*, *A. altissima*, *P. orientalis*, and *P. davidiana*, followed by *P. tomentosa*, *A. persica*, and *S. babylonica. M. alba* demonstrated the least attraction for *C. cunea* based on its HIPVs. Consequently, *M. alba*, which is the most preferred host of *H. cunea*, is the least attractive to *C. cunea*. This phenomenon may elucidate why *M. alba* is the most preferred host of *H. cunea*, despite not being its optimal nutritional host for growth and development. *A. persica* and *S. babylonica* exhibited weak attraction to *C. cunea*, yet *H. cunea* still did not prefer these plant species, potentially due to their poor nutritional value for growth and development. Therefore, it appears that *H. cunea* engages in a trade-off between performance and survival, preferring a host plant with suboptimal nutrition in favor of mitigating parasitism. A similar correlation between plant quality and larval parasitism was observed in field investigations. *H. cunea* larvae reared on higher-quality host plants also showed a greater proportion of mortality attributable to parasitism [39].

To identify the biologically active HIPVs, we analyzed the leaf volatiles of eight different plant species before and after *H. cunea* feeding. GC-EAD, followed by GC-MS analysis, identified six electrophysiologically active saturated hydrocarbons (undecane, tridecane, tetradecane, hexadecane, nonadecane, and heneicosane) [40].

HIPVs comprise a variety of divergent VOCs, including alkanes, alkenes, aldehydes, alcohols, ketones, ethers, esters, and carboxylic acids [40]. The HIPVs identified in this study are all alkanes. We hypothesize that in addition to these alkanes, there might be a variety of other compounds present. In subsequent research, we will continue to investigate other active compounds that guide host preference. The alkanes identified in this study have also been observed in other studies as active components in HIPVs. Undecane has been demonstrated to be a HIPV from immature fruits of Java plum *Syzygium cuminii* [41]. Tetradecane, as a major bioactive component of HIPVs produced by fruits, has been shown to regulate the behavioral response of the egg–pupal endoparasitoid *Fopius arisanus* [42]. The parasitoid *Microplitis similis* selection of cabbage was positively correlated with the release of hexadecane, as one of the HIPV components to defend against *Spodoptera litura* [43]. *Cucurbita maxima* damaged by *Aulacophora foveicollis* released nonadecane [44]. *Nicotiana tabacum*, infested by sap-sucking aphid *Myzus persicae* Sulzer, produced heneicosane as a HIPV [45].

Of the six compounds, tridecane was not detected in only one host plant, *M. alba*. In several studies, tridecane has been demonstrated to be a herbivore-induced plant volatile (HIPV) that attracts natural enemies. For instance, tobacco plants (*Nicotiana tabacum* L.), infested by sap-sucking aphids (*Myzus persicae* Sulzer) produce tridecane and other compounds as HIPVs [45]. The Asian egg parasitoid *Trissolcus japonicas* utilizes tridecane as the kairomone of the brown marmorated stink bug, *Halyomorpha haly,* for short-range host location [46]. Additionally, the predaceous minute pirate bug, *Orius insidiosus* (Say), employs tridecane as a kairomone to locate the brown marmorated stink bug [47]. Tridecane is also a component of the alarm volatiles of stinkbugs (*Tessaratoma papillosa*), released when stinkbugs are disturbed or irritated [48]. Furthermore, tridecane is a component of the pygidial gland of larval secretions, playing a role in chemical defense against predators such as ants [49].

Our investigation revealed opposite responses to HPIVs by the herbivore (*H. cunea*) and its parasitoid (*C. cunea*). In isolation, tridecane, a prominent HPIV in seven of the eight host plants, did not elicit any behavioral responses, either attraction or repellency, in *H. cunea* or *C. cunea*. However, when combined with other HPIV compounds, the mixture that included tridecane significantly attracted *C. cunea* and repelled *H. cunea*. *M. alba*, the only host plant that did not emit tridecane in its HPIVs, did not attract the wasps and was preferred by the moth over the other seven host plants. Most insect olfactory receptors require cooperative binding of multiple volatile molecules to trigger responses. A single chemical is typically insufficient to activate the olfactory receptor channel [50]. Thus, when acting alone, tridecane exhibits insufficient binding affinity, whereas other compounds in the mixture may provide auxiliary binding sites that collectively reach the receptor activation threshold.

It is generally accepted that natural enemies rely on HIPVs for long-range localization of an appropriate habitat [51] and the host insect’s volatiles for the short-range location of the host [52,53]. In this investigation, we identified tridecane, hexadecane, nonadecane, and heneicosane as HIPVs that attracted *C. cunea* wasps. In a previous study, we identified six compounds (1-dodecene, hexadecane, heptadecane, nonadecane, heneicosane, and heptacosane) emitted from the pupae of three lepidopteran species, including *H. cunea*, that serve as hosts for *C. cunea* [54]. It is particularly noteworthy that both behaviorally active blends (from plant hosts and insect hosts) comprise hydrocarbons with similar characteristics. We therefore postulate that the semiochemicals released by plants and those released by host insects may be chemically related and thus perhaps represent the convergence of tritrophic systems on suites of related compounds. Identifying common semiochemicals may be a more efficient strategy for *C. cunea*, corresponding to the parasitoid wasp’s limited neural capacity [55]. The responses of the mated female *H. cunea* to different plant leaves pre-infested by *H. cunea* will be examined in subsequent research, which is crucial for developing practical applications in the field ecosystem. It is also of interest whether the leaf volatiles (HIPVs) before and after feeding by other herbivores consist of the same emissions, or if *H. cunea* elicits specific HIPVs.

## 5. Conclusions

The main findings of this study are summarized as follows: The preferred host plant of female *H. cunea* was *M. alba*. However, compared with other host plants, *M. alba* was not the optimal nutritional host for *H. cunea* growth and development. Compared with other host plants, *M. alba* was the least attractive to the parasitoid *C. cunea*, a natural enemy of *H. cunea*. GC-EAD and GC-MS analyses identified six volatiles as HIPVs of different host plants fed upon by *H. cunea*. These HIPVs elicited EAD responses in the antennae of female *C. cunea*. Tridecane was emitted by seven of eight host plant species, but not by *M. alba*. Tridecane alone did not attract or repel either *C. cunea* or *H. cunea*, but when combined with other HPIVs, the mixture significantly attracted *C. cunea* and repelled *H. cunea*. *M. alba* represents “enemy-less space” for *H. cunea*. By preferring *M. alba*, which does not emit tridecane, *H. cunea* effectively avoids parasitism by *C. cunea*, which is highly attracted to HIPVs that contain tridecane.

## Figures and Tables

**Table 1:** Compounds were identified by GC peak enhancement using authentic standards on an Agilent DB-5 column.

	Compound	Retention Time	Emission Rate (ng/min from 1 g Leaves)							
			***M. alba***	***P. davidiana***	***P. orientalis***	***A. altissima***	***S. babylonica***	***A. persica***	***P. tomentosa***	***F. chinensis***
1	Undecane	8.85	31.48	9.02	17.87	44.36	16.52	15.70	17.51	19.07
2	Tridecane	11.43	N	30.76	30.26	30.40	4.98	9.12	21.10	27.72
3	Tetradecane	12.60	22.85	15.11	11.41	19.25	13.70	19.91	14.11	35.97
4	Hexadecane	14.86	20.63	15.98	23.20	42.31	13.50	21.01	17.21	13.36
5	Nonadecane	17.85	17.23	8.91	21.80	24.60	13.68	15.68	13.62	14.68
6	Heneicosane	24.81	17.17	11.47	18.57	20.24	13.42	13.95	13.56	22.73

## Data Availability

The original contributions presented in this study are included in the article material. Further inquiries can be directed at the corresponding author(s).

## References

[1] Biere A., Elzinga J.A., Honders S.C., Harvey J.A. (2002). A plant pathogen reduces the enemy-free space of an insect herbivore on a shared host plant. Proc. R. Soc. B-Biol. Sci..

[2] Singer M.S., Rodrigues D., Stireman J.O., Carriere Y. (2004). Roles of food quality and enemy-free space in host use by a generalist insect herbivore. Ecology.

[3] Hu X.Y., Su S.L., Liu Q.S., Jiao Y.Y., Peng Y.F., Li Y.H., Turlings T.C.J. (2020). Caterpillar-induced rice volatiles provide enemy-free space for the offspring of the brown planthopper. Elife.

[4] Singer M.S., Stireman J.O. (2003). Does anti-parasitoid defense explain host-plant selection by a polyphagous caterpillar?. Oikos.

[5] Jeffries M.J., Lawton J.H. (1984). Enemy free space and the structure of ecological communities. Biol. J. Linn. Soc..

[6] Price P.W., Fernandes G.W., Waring G.L. (1987). Adaptive nature of insect galls. Environ. Entomol..

[7] Lill J.T., Marquis R.J., Ricklefs R.E. (2002). Host plants influence parasitism of forest caterpillars. Nature.

[8] Mulatu B., Applebaum S.W., Coll M. (2004). A recently acquired host plant provides an oligophagous insect herbivore with enemy-free space. Oikos.

[9] Alhmedi A., Bylemans D., Bangels E., Belien T. (2021). Cultivar-mediated effects on apple-*Dysaphis plantaginea* interaction. J. Pest Sci..

[10] Ohsaki N., Sato Y. (1994). Food plant choice of pieris butterflies as a trade-off between parasitoid avoidance and quality of plants. Ecology.

[11] Vidal M.C., Murphy S.M. (2018). Bottom-up vs. top-down effects on terrestrial insect herbivores: A meta-analysis. Ecol. Lett..

[12] Englishloeb G.M., Brody A.K., Karban R. (1993). Host-plant-mediated interactions between a generalist folivore and its tachinid parasitoid. J. Anim. Ecol..

[13] Williams I.S., Jones T.H., Hartley S.E. (2001). The role of resources and natural enemies in determining the distribution of an insect herbivore population. Ecol. Entomol..

[14] Murphy S.M., Lill J.T., Bowers M.D., Singer M.S. (2014). Enemy-Free Space for Parasitoids. Environ. Entomol..

[15] Ji R., Xie B.Y., Li X.H., Gao Z.X., Li D.M. (2003). Research progress on the invasive species, *Hyphantria cunea*. Entomol. Knowl..

[16] Gao B.J., Du J., Gao S.H., Liu J.X. (2010). Genetic diversity and differentiations of fall webworm (*Hyphantria cunea*) populations. Sci. Silvae Sin..

[17] Zhang X.X., Wang Z.J. (2009). Research progress on the *Hyphantria cunea* (Drury) of alien invasive species. J. Anhui Agric. Sci..

[18] Yang Z.Q. (1989). A new genus and species of Eulophidae (Hymenoptera: Chalcidoidea) parasitizing *Hyphantria cunea* (Drury) (Lepidoptera: Arctiidae) in China. Entomotaxonomia.

[19] Yang Z.Q. (1995). Anatomy of internal reproductive system of *Chouioia cunea* (Hymenoptera, Chalcidoidea, Eulophidae). Sci. Silvae Sin..

[20] Yang Z.Q. (2004). Advance in bio-control researches of the important forest insect pests with natural enemies in China. Chin. J. Biol. Control.

[21] Yang Z.Q., Wang X.Y., Wei J.R., Qu H.R., Qiao X.R. (2008). Survey of the native insect natural enemies of *Hyphantria cunea* (Drury) (Lepidoptera: Arctiidae) in China. Bull. Entomol. Res..

[22] Zheng Y.N., Qi J.Y., Sun S.H., Yang C.C. (2012). Advance in research of *Chouioia cunea* Yang (Hymenoptera: Eulophidade) and its biocontrol application in China. Chin. J. Biol. Control.

[23] Pan L., Gao W., Liu X., Qin D., Zhang T., Ren R., Zhang W., Sun M., Gao C., Bai P. (2023). Parasitoids as taxonomists: How does the parasitoid *Chouioia cunea* distinguish between a host and a non-host?. Pest. Manag. Sci..

[24] Li L.S., Yuan Y.F., Wu L., Chen M. (2018). Effects of host plants on the feeding behavior and detoxification enzyme activities in *Hyphantria cunea* (Lepidoptera: Arctiidae) larvae. Acta Entomol. Sin..

[25] Hu D.Q., Li R., Song X.X. (2019). Research on feeding habits of *Hyphantria cunea* larvae in Qingdao area. Heilongjiang Agric. Sci..

[26] Wang F., Zhang L.H., Han H.Z., Wang X.L., Zhang Y., Li S.H. (2020). Feeding preference of *Hyphantria cunea* Drury larvae to common garden plants in Suqian area. J. Henan Agric. Univ..

[27] Costa A., Reeve J.D. (2011). Olfactory Experience Modifies Semiochemical Responses in a Bark Beetle Predator. J. Chem. Ecol..

[28] Lo Giudice D., Riedel M., Rostas M., Peri E., Colazza S. (2011). Host Sex Discrimination by an Egg Parasitoid on Brassica Leaves. J. Chem. Ecol..

[29] Mouratidis A., Vacas S., Herrero J., Navarro-Llopis V., Dicke M., Tena A. (2021). Parasitic wasps avoid ant-protected hemipteran hosts via the detection of ant cuticular hydrocarbons. Proc. R. Soc. B-Biol. Sci..

[30] Piesik D., Wenda-Piesik A., Krasinska A., Wrzesinska D., Delaney K.J. (2016). Volatile organic compounds released by *Rumex confertus* following *Hypera rumicis* herbivory and weevil responses to volatiles. J. Appl. Entomol..

[31] Gantner M., Najda A., Piesik D. (2019). Effect of phenolic acid content on acceptance of hazel cultivars by filbert aphid. Plant Prot. Sci..

[32] Piesik D., Wenda-Piesik A. (2015). *Sitophilus granarius* responses to blends of five groups of cereal kernels and one group of plant volatiles. J. Stored Prod. Res..

[33] Zhu G., Pan L., Zhao Y., Zhang X., Wang F., Yu Y., Fan W., Liu Q., Zhang S., Li M. (2017). Chemical investigations of volatile kairomones produced by *Hyphantria cunea* (Drury), a host of the parasitoid *Chouioia cunea* Yang. Bull. Entomol. Res..

[34] Awater-Salendo S., Hilker M., Fürstenau B. (2023). Kairomone-induced changes in foraging activity of the larval ectoparasitoid *Holepyris sylvanidis* are linked with an increased number of male parasitoid offspring. Front. Ecol. Evol..

[35] Liu C.M., Xu F.Y., Jiang J.H., Wu J.Y., Wang M.M., Li Y., Wang Y.H., Wei D.X. (2013). Effects of main tree species in northern Jiangsu on occurrence and larval growth of *Hyphantria cunea*. J. Jiangsu For. Sci. Technol..

[36] Qin X.B., Li D.J., Shao W.H., Li Z.P., Jiang C.P., Qu H.R., Wang C.Z. (2000). Problems and suggestions on the control of *Hyphantria cunea* in Shandong Province. For. Pest Dis..

[37] Yu S.W. (2016). Oviposition habit and host selection of *Hyphantria cunea* in Langfang. Mod. Rural. Sci. Technol..

[38] Chen H., Su H.H., Zhang S., Jing T.X., Liu Z., Yang Y.Z. (2021). The Effect of Mirid Density on Volatile-Mediated Foraging Behaviour of *Apolygus lucorum* and *Peristenus spretus*. Insects.

[39] Murphy S.M., Loewy K.J. (2015). Trade-offs in host choice of an herbivorous insect based on parasitism and larval performance. Oecologia.

[40] D’Alessandro M., Turlings T.C.J. (2006). Advances and challenges in the identification of volatiles that mediate interactions among plants and arthropods. Analyst.

[41] Jayanthi P.D.K., Subramoniam A., Kumar P.S., Jayanthimala B.R., Rekha A. (2021). Do conspecific herbivores track resource depletion through host phenology-specific HIPVs?. Curr. Sci..

[42] Cai P.M., Song Y.Z., Huo D., Lin J., Zhang H.M., Zhang Z.H., Huang F.M., Xiao C.M., Ji Q.E. (2020). Chemical cues mediating behavioral and electrophysiological responses of *Fopius Arisanus* (Hymenoptera: Braconidae): The Role Of Herbivore-Induced Plant Volatiles. Appl. Ecol. Environ. Res..

[43] Du Y.W., Shi X.B., Zhao L.C., Yuan G.G., Zhao W.W., Huang G.H., Chen G. (2022). Chinese Cabbage Changes Its Release of Volatiles to Defend against *Spodoptera litura*. Insects.

[44] Bhowmik B., Chakraborti U., Mandal A., Paul B., Bhadra K. (2022). Attraction of *Aulacophora foveicollis* Lucas (Coleoptera: Chrysomelidae) to Host Plant *Cucurbita maxima* Duchesne (Cucurbitaceae) Volatiles. Agronomy.

[45] Song Y.Z., Guo Y.Q., Cai P.M., Chen W.B., Liu C.M. (2021). Alteration of volatile chemical composition in tobacco plants due to green peach aphid (*Myzus persicae sulzer*) (Hemiptera: Aphididae) feeding. Appl. Ecol. Environ. Res..

[46] Malek R., Kaser J.M., Anfora G., Ciolli M., Khrimian A., Weber D.C., Hoelmer K.A. (2021). *Trissolcus japonicus* foraging behavior: Implications for host preference and classical biological control. Biol. Control.

[47] Fraga D.F., Parker J., Busoli A.C., Hamilton G.C., Nielsen A.L., Rodriguez-Saona C. (2017). Behavioral responses of predaceous minute pirate bugs to tridecane, a volatile emitted by the brown marmorated stink bug. J. Pest Sci..

[48] Zhang Z.M., Wu W.W., Li G.K. (2009). Study of the Alarming Volatile Characteristics of *Tessaratoma papillosa* Using SPME-GC-MS. J. Chromatogr. Sci..

[49] Gasch T., Vilcinskas A. (2014). The chemical defense in larvae of the earwig *Forficula auricularia*. J. Insect Physiol..

[50] Zhao J., Chen A.Q., Ryu J., Del Mármol J. (2024). Structural basis of odor sensing by insect heteromeric odorant receptors. Science.

[51] Turlings T.C.J., Wäckers F. (2004). Recruitment of Predators and Parasitoids by Herbivore-Injured Plants.

[52] Vet L.E.M., Dicke M. (1992). Ecology of infochemical use by natural enemies in a tritrophic context. Annu. Rev. Entomol..

[53] van Alphen J.J.M., Jervis M.A. (1996). Insect Natural Enemies: Practical Approaches to Their Study and Evolution..

[54] Li M., Yang Y.X., Yao Y.H., Xiang W.F., Han J.Y., Wang Y.H., Bai P.H., Wang J., Zhu G.P., Man L. (2020). Isolation and identification of attractants from the pupae of three lepidopteran species for the parasitoid *Chouioia cunea* Yang. Pest Manag. Sci..

[55] Schoonhoven L.M., Van Loon J.J.A.J.J.L., Dicke M. (2005). Insect–Plant Biology.

